# Astrocyte Activation and the Calcineurin/NFAT Pathway in Cerebrovascular Disease

**DOI:** 10.3389/fnagi.2018.00287

**Published:** 2018-09-21

**Authors:** Susan D. Kraner, Christopher M. Norris

**Affiliations:** ^1^Sanders-Brown Center on Aging, University of Kentucky College of Medicine, Lexington, KY, United States; ^2^Department of Pharmacology and Nutritional Sciences, University of Kentucky College of Medicine, Lexington, KY, United States

**Keywords:** vascular contributions to cognitive impairment and dementia, Ca^2+^, glia, excitotoxicity, Alzheimer’s disease

## Abstract

Calcineurin (CN) is a Ca^2+^/calmodulin-dependent protein phosphatase with high abundance in nervous tissue. Though enriched in neurons, CN can become strongly induced in subsets of activated astrocytes under different pathological conditions where it interacts extensively with the nuclear factor of activated T cells (NFATs). Recent work has shown that regions of small vessel damage are associated with the upregulation of a proteolized, highly active form of CN in nearby astrocytes, suggesting a link between the CN/NFAT pathway and chronic cerebrovascular disease. In this Mini Review article, we discuss CN/NFAT signaling properties in the context of vascular disease and use previous cell type-specific intervention studies in Alzheimer’s disease and traumatic brain injury models as a framework to understand how astrocytic CN/NFATs may couple vascular pathology to neurodegeneration and cognitive loss.

## Introduction

Cerebrovascular pathology is one of the leading causes of cognitive loss and mortality. While stroke is usually the most devastating form of cerebrovascular disease, other forms of vascular damage and dysfunction including microinfarcts, microhemorrhages, cerebral amyloid angiopathy and cerebral hypoperfusion are more insidious and can lead to chronic and progressive cognitive loss, especially in aged individuals. These vascular contributions to cognitive impairment and dementia (VCID) are the second leading cause of dementia, behind Alzheimer’s disease, and frequently co-exist with other neurodegenerative conditions (O’Brien et al., 2003). Importantly, VCID comorbidities appear to interfere with the treatment of Alzheimer’s disease-related functional deficits in animal models (Weekman et al., 2016), highlighting the need to understand the cellular mechanisms that link vascular dysfunction to neurodegeneration and impaired cognition (Snyder et al., 2015; Horsburgh et al., 2018).

Brain ischemia results when stroke or other forms of VCID block the blood supply to parts of the brain, resulting in depletion of oxygen and glucose. This depletion rapidly exhausts the energy production of neural cells and their ability to maintain the normal balance of ions across cellular membranes, thus causing excitotoxicity and Ca^2+^ overload, among other adverse effects (Choi, 1988; Horst and Postigo, 1996; Szydlowska and Tymianskia, 2010). Ca^2+^ overload originates from a variety of sources and directly affects numerous intracellular signaling cascades, many of which have been explored as potential treatment targets for stroke and other forms of cerebrovascular disease (Harris et al., 1982; Infeld et al., 1999; Ray, 2006; Mattson, 2007; Rostas et al., 2017; Wu and Tymianski, 2018). In most cases, Ca^2+^-signaling pathways have been investigated in neurons, which are the primary target of excitotoxic damage. In the following Mini Review article, we will discuss the importance of the Ca^2+^/calmodulin dependent protein phosphatase, calcineurin (CN) and its dysregulation in astrocytes as a pathological mechanism and potential target for neurodegeneration and cognitive loss due to cerebrovascular damage.

## CN Dysregulation in Stroke Models

CN, or protein phosphatase 3, is the only phosphatase in mammals that is directly activated by Ca^2+^/calmodulin. CN consists of a catalytic subunit (PPP3CA) and a Ca^2+^ binding regulatory subunit (PPP3R1). When cellular Ca^2+^ levels are low, the phosphatase activity of CN is held in check by an autoinhibitory domain located near the C terminus of the catalytic subunit. The interaction of Ca^2+^ with the CN regulatory subunit and calmodulin leads to a physical interaction between the CN catalytic subunit and Ca^2+^/calmodulin, which, in turn, displaces the AID and frees the catalytic core from inhibition. When cellular Ca^2+^ levels fall, calmodulin is released from the catalytic subunit and AID-mediated inhibition of phosphatase activity is reinstated (Klee et al., 1998; Aramburu et al., 2000). In healthy nervous tissue, CN provides an essential mechanism for bidirectional synaptic plasticity through the induction and maintenance of activity-dependent synaptic depression (Mansuy, 2003). In this capacity, CN is widely thought to link Ca^2+^ signaling to several forms of learning and memory, including extinction learning (Baumgärtel et al., 2008; de la Fuente et al., 2011; Rivera-Olvera et al., 2018). However, due to its exquisite sensitivity to Ca^2+^, CN is also frequently identified as a central player in numerous deleterious or maladaptive processes arising from Ca^2+^ overload and/or dysregulation (Uchino et al., 2008; Mukherjee and Soto, 2011; Reese and Taglialatela, 2011; Furman and Norris, 2014; Sompol and Norris, 2018).

Large and/or sustained surges in Ca^2+^ can lead to calpain or caspase-mediated proteolytic disruption of the CN AID (Wang et al., 1989; Wu et al., 2004), which partially and irreversibly uncouples CN from Ca^2+^, resulting in constitutive phosphatase activity. Several acute and chronic neurodegenerative conditions are associated with the generation of high activity CN proteolytic fragments (ΔCN), thus perpetuating de-phosphorylation of the myriad of CN targets (Norris, 2014). Hypoxic/ischemic insults appear to be particularly effective at triggering the proteolysis of CN from its full length highly-regulated form (60 kDa), to high activity fragments (ΔCN) ranging in size from 45 to 57 kDa (Shioda et al., 2006, 2007; Rosenkranz et al., 2012). Conversely, blockade of CN typically provides considerable neuroprotection during ischemia and other adverse consequences of cerebrovascular damage. For instance, the CN inhibiting immunosuppressant drug, tacrolimus (or FK506), has been shown to reduce infarct size (Sharkey and Butcher, 1994; Butcher et al., 1997), suppress neuroinflammation (Zawadzka and Kaminska, 2005) and promote recovery of function (Sharkey et al., 1996) in middle cerebral artery occlusion models of ischemic stroke. More recently, a CN modulatory protein, known as regulator of CN (RCAN), was found to favorably affect the pathogenesis of stroke *in vivo* and hypoxia *in vitro* using both gene overexpression and knockout approaches (Brait et al., 2012; Sobrado et al., 2012). Together, these results suggest that CN proteolysis (hyperactivation) is not only a biomarker, but also an important mediator, of neurodegeneration resulting from vascular damage.

## NFATs

The exact mechanisms through which CN acts are complex and multifaceted. CN has a broad and diverse range of substrates, many of which have been implicated as downstream targets in CN-mediated cellular dysfunction and neurotoxicity (Uchino et al., 2008; Mukherjee and Soto, 2011; Reese and Taglialatela, 2011; Furman and Norris, 2014). Perhaps the best characterized substrate of CN is the nuclear factor of activated T cells (NFATs), a transcription factor related to NFκB/Rel-family proteins (Rao et al., 1997). There are four CN-dependent NFAT family members (NFATs 1–4), all of which are expressed in nervous tissue (Nguyen and Di Giovanni, 2008; Vihma et al., 2008). NFATs reside in the cytosol in their resting state, but upon de-phosphorylation by CN, they translocate to the nucleus where they can activate or suppress numerous gene expression programs linked to immune/inflammatory signaling, Ca^2+^ regulation, and cell survival, among other things (Im and Rao, 2004). NFAT isoforms have different cellular distributions inside and outside of the nervous system (Horsley and Pavlath, 2002; Abdul et al., 2010) and appear to engage in both overlapping and distinct transcriptional programs through interactions with multiple other transcription factor families (Rao et al., 1997; Im and Rao, 2004; Wu et al., 2006). Of the four isoforms, NFATs 1 and 4 seem to show a greater bias for glial cells where they respond to many different kinds of inflammatory factors and other noxious stimuli, including blood derived factors (Canellada et al., 2008; Sama et al., 2008; Abdul et al., 2009; Nagamoto-Combs and Combs, 2010; Serrano-Pérez et al., 2011; Neria et al., 2013; Furman et al., 2016; Manocha et al., 2017; Sompol et al., 2017).

## Hyperactive Astrocytic CN/NFAT Signaling: Biomarker for Vascular Damage?

**Figure 1:**
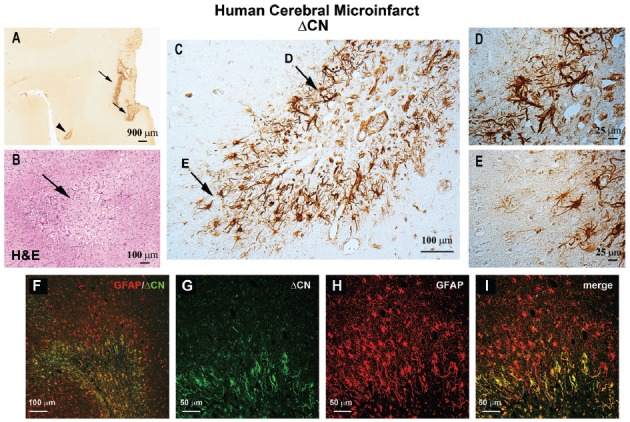
ΔCN is intensely expressed in activated astrocytes surrounding microinfarcts in human neocortex. **(A)** Representative low magnification photomicrograph from superior and middle temporal gyrus (SMTG) of a 90 year old human subject with multiple microinfarcts, but little-to-no Alzheimer’s pathology (Braak stage II) ΔCN labeling is present around several microinfarcts (arrows and arrowhead). **(B)** Serial section through STMG stained by H&E to confirm the presence of microinfarcts. The image shown is a high magnification of the region denoted by the arrowhead in Panel **(A)**. **(C)** High power photomicrograph of the region in (**A**; arrowhead) showing intense ΔCN antibody labeling of astrocytes. Higher magnification of the areas denoted by arrows are shown in panels **(D,E)**. **(F)** Merged confocal micrograph showing the colocalization of ΔCN (green) with GFAP around a microinfarct in human SMTG (red). **(G–I)** High magnification images of the infarct in Panel **(F)** shown in individual channels **(G,H)** and merged **(I)**. Co-localization of ΔCN with GFAP was most extensive in the region immediately adjacent to the infarct. From Pleiss et al. (2016) used with permission.

Astrocytic CN/NFAT signaling may provide, and give rise to, useful biomarkers for cerebrovascular damage. One of the most striking changes in CN/NFAT expression following CNS injury and disease is strong and selective expression in subsets of activated astrocytes (Hashimoto et al., 1998; Norris et al., 2005; Celsi et al., 2007; Serrano-Pérez et al., 2011; Lim et al., 2013; Neria et al., 2013; Furman et al., 2016; Pleiss et al., 2016; Sompol et al., 2017). For instance, the NFAT4 isoform, which is weakly expressed in healthy nervous tissue, appears at elevated levels in many activated astrocytes following kainic acid lesions, cortical stab wounds and controlled cortical contusion injuries (Serrano-Pérez et al., 2011; Neria et al., 2013; Furman et al., 2016). NFAT4 expression in a mouse model of Alzheimer’s disease also exhibited extensive co-localization with activated astrocytes, increasing directly in proportion to the expression of GFAP (Sompol et al., 2017). Using a custom antibody to CN, based on calpain-dependent cleavage sites, our lab recently observed intense labeling of a 45–48 kDa ΔCN fragment in activated astrocytes surrounding microinfarcts in human neocortex (Pleiss et al., 2016). Labeling for ΔCN was very faint throughout most brain areas examined, but increased dramatically in GFAP-positive astrocytes around the periphery of the lesion (Figure 1). These observations suggest considerable molecular heterogeneity in astrocytes depending on distance from vascular injury, consistent with studies in other injury/disease models (Zamanian et al., 2012; Itoh et al., 2018).

Several outstanding issues regarding the relationship between astrocytic CN/NFAT and microinfarcts require further clarification. Presently, it is unknown whether CN/NFAT alterations occur immediately following microinfarct induction, or are more characteristic of chronic changes that arise with the formation of glial scars. The molecular phenotype of ΔCN-positive astrocytes has also yet to be elucidated. In primary neural cultures, forced overexpression of ΔCN in astrocytes induces the expression of numerous transcripts associated with morphogenesis and immune response (Norris et al., 2005). Studies are presently underway in our lab to determine the time course of ΔCN expression in photothrombosis models of microinfarct pathology (Risher et al., 2010; Masuda et al., 2011; Summers et al., 2017; Underly and Shih, 2017) and to determine if endogenous expression of ΔCN is associated with transcriptional changes, reminiscent of forced overexpression studies.

It deserves noting that many of the transcripts induced by CN/NFAT activity in glial cells, and in other cell types, encode releasable factors, such as cytokines and chemokines (Norris et al., 2005; Canellada et al., 2008; Sama et al., 2008; Nagamoto-Combs and Combs, 2010; Neria et al., 2013). Given the intimate structural and functional interactions between astrocytes and cerebral blood vessels, it seems likely that many CN/NFAT-dependent factors released from activated astrocytes could find their way into the bloodstream near regions of vascular damage. Presence of these factors (or ΔCN itself) in blood could then be used as potential biomarkers for the presence of microinfarcts or other forms of vascular pathology. Indeed, given the insidious nature of microinfarcts, the identification of peripheral biomarkers would be most helpful for diagnostic and/or prognostic screening purposes. Of course, additional research will be necessary to assess these possibilities.

## Functional Impact of CN Signaling in Activated Astrocytes

Astrocyte activation is a complex process associated with both neuroprotective and deleterious consequences for surrounding nervous tissue (Khakh and Sofroniew, 2015; Pekny et al., 2016; Verkhratsky et al., 2016). The increased expression of CN/NFAT components in astrocytes associated with vascular pathology may offer important targets that could be exploited for determining the functional impact of these cells. Overexpression of ΔCN in hippocampal astrocytes of intact healthy adult rats causes reduced synaptic strength and hyperexcitability in nearby neurons, which is consistent with other studies linking activated astrocytes with impaired neuronal connectivity in acute injury models (Wilhelmsson et al., 2004). In contrast, astrocytic expression of ΔCN has also been found to reduce amyloid pathology and improve cognitive function in mouse models of Alzhieimer’s disease, consistent with other reports that have found protective roles of activated astrocytes in neurodegenerative conditions (Okada et al., 2006; Kraft et al., 2013; Wanner et al., 2013; Tyzack et al., 2014). Whether CN gives rise to beneficial or detrimental processes may depend critically on the presence of different activating factors and/or the recruitment of different transcription factor families (Furman and Norris, 2014). For instance, the pro-inflammatory cytokine TNF was shown to trigger the association of CN with the transcription factors NFκB and FOXO3, which, in turn, induced pro-inflammatory responses for promoting neurodegeneration (Fernandez et al., 2012, 2016). In contrast, CN stimulation by the insulin-like growth factor (IGF-I), has been proposed to mediate neuroprotective responses of activated astrocytes via interactions between NFκB and PPARγ (Fernandez et al., 2012).

Blockade of CN interactions with NFAT transcription factors, using the peptide VIVIT, has been associated with many beneficial effects in cell culture and intact animal models of neurodegeneration. VIVIT mimics the CN-binding PxIxIT motif found in the regulatory region of NFATs 1–4 (Aramburu et al., 1999). When delivered to numerous cell types, VIVIT prevents CN from binding to NFATs and therefore inhibits NFAT nuclear localization, without inhibiting CN catalytic activity *per se*. Expression of VIVIT in hippocampal astrocytes, using adeno-associated virus (AAV) vectors equipped with the human GFAP promoter Gfa2 (Lee et al., 2008), improved synaptic strength and/or normalized synaptic plasticity in animal models of Alzheimer’s disease and traumatic brain injury (Furman et al., 2012, 2016; Sompol et al., 2017). Where tested, AAV-Gfa2-VIVIT delivery to the hippocampus also improved hippocampal-dependent cognitive function (Furman et al., 2012; Sompol et al., 2017). In primary neural cultures, VIVIT prevented the loss of astrocyte-enriched glutamate transporters, primarily GLT1, in response to pro-inflammatory cytokines and oligomeric Aβ, leading to reduced extracellular glutamate levels, reduced neuronal excitability and greater neuronal survival (Sama et al., 2008; Abdul et al., 2009). VIVIT similarly restored GLT1 levels in intact 5xFAD mice—an aggressive mouse model for Alzheimer’s disease (Sompol et al., 2017). Mice treated with AAV-Gfa2-VIVIT showed greater GLT1 expression, measured via immunofluorescent microscopy and Western blot. VIVIT-treated 5xFAD mice also exhibited fewer and shorter-duration spontaneous glutamate transients (measured *in vivo*), healthier neurite morphology, reduced synaptic hyperexcitability, and normalized NMDA-to-AMPA receptor activity ratios (Sompol et al., 2017). Together, these observations suggest that hyperactive CN/NFAT signaling underlies a neurotoxic activated astrocyte phenotype characterized by glutamate dysregulation and excitotoxicity.

Interestingly, many of the same telltale signs of glutamate toxicity, including a loss of GLT1 and neuronal hyperactivity, have been noted in experimental models of ischemia and stroke (Maragakis and Rothstein, 2004; Soni et al., 2014). Moreover, glutamate dysregulation would not only influence the behavior and viability of surrounding neurons, but may also be expected to negatively affect the cerebrovascular unit as well. For instance, functional knockdown of GLT1 in otherwise healthy animals can lead to reduced cerebral blood flow and/or impaired neurovascular coupling (Petzold et al., 2008). Other work has shown that hyperexcitable neural networks and/or excitotoxic insults compromise the structural integrity of vascular endothelial cells and perivascular astrocyte endfeet, and precipitate blood brain barrier (BBB) leakage (Bolton and Perry, 1998; Parathath et al., 2006; Alvestad et al., 2013; Gondo et al., 2014; Ryu and McLarnon, 2016) leading to perivascular and parenchymal neuroinflammation.

## Summary and Future Directions

**Figure 2:**
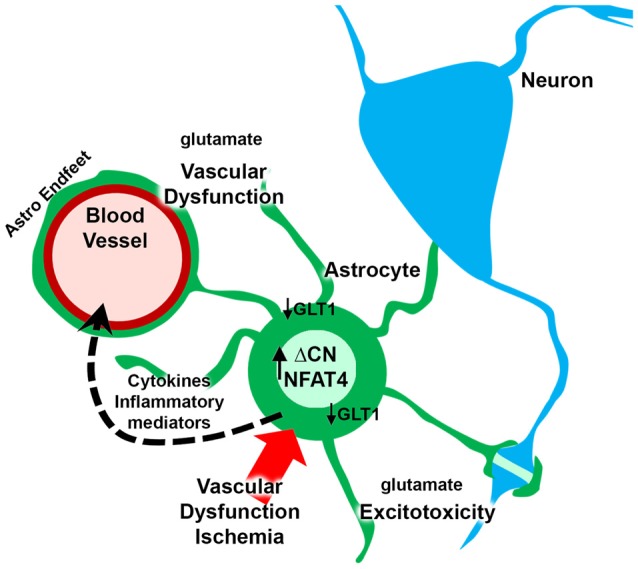
Putative role for astrocytic CN/nuclear factor of activated T cell (NFAT) in vascular dysfunction and neurodegeneration. Ischemia arising from vascular degeneration or disruption leads to increased expression of ΔCN and hyperactivation of NFAT4 in astrocytes. The CN/NFAT pathway induces numerous cytokines and other inflammatory mediators linked to neuroinflammation. Some of these factors may target blood vessels, leading to perivascular inflammation. CN/NFAT signaling also leads to the *downregulation* of GLT1 glutamate transporters resulting in *elevated* extracellular glutamate levels. Glutamate causes excitotoxicity at synaptic connections and disrupts astrocyte endfeet and/or blood brain barrier (BBB) integrity, leading to further vascular dysfunction and/or degeneration.

Cerebrovascular pathology is one of the leading causes of dementia and a frequently identified comorbid factor in many neurologic diseases, such as Alzheimer’s disease. Numerous studies have reported a role for CN hyperactivity in the pathophysiologic sequelae coupling vascular disruption and damage to neuronal death and cognitive loss. Mounting evidence suggests that CN/NFAT signaling may play a particularly important role in neural changes that arise with astrocyte activation in many different neurodegenerative diseases, including cerebrovascular disease. However, no studies to date have tested the specific involvement of astrocytic CN/NFAT signaling in either global ischemia models, models characterized by localized damage to microvessels, or in models that develop chronic vascular inflammation and microhemhorrages. Based on the observations discussed above, we hypothesize that acutely and chronically developing vascular damage will lead to the activation of astrocytes and hyperactivation of CN/NFAT signaling (Figure 2). In this scenario, increased CN/NFAT activity would lead to the induction and release of numerous immune/inflammatory factors and/or to the dysregulation of astrocytic glutamate uptake, resulting in impaired synaptic function, excitotoxicity, impaired neuronal viability and neuroinflammation. These deleterious actions, could, in turn, promote further vascular damage and inflammation and hasten neurodegeneration and cognitive loss as part of vicious positive feedback cycle. Of course, this hypothesis will require extensive testing using astrocyte-specific targeting strategies in experimental models of stroke and/or VCID.

## Author Contributions

SK and CN researched and wrote this manuscript.

## Conflict of Interest Statement

The authors declare that the research was conducted in the absence of any commercial or financial relationships that could be construed as a potential conflict of interest.
